# Optimal controlling of anti-TGF-$\beta$ and anti-PDGF medicines for preventing pulmonary fibrosis

**DOI:** 10.1038/s41598-023-41294-z

**Published:** 2023-09-12

**Authors:** Fatemeh Bahram Yazdroudi, Alaeddin Malek

**Affiliations:** Department of Applied Mathematics, Faculty of Mathematical Sciences, Tarbiat Modares University, Tehran, Iran

**Keywords:** Applied mathematics, Computational biology and bioinformatics, Predictive medicine

## Abstract

In the repair of injury, some transforming growth factor-$\beta$s (TGF-$\beta$s) and platelet-derived growth factors (PDGFs) bind to fibroblast receptors as ligands and cause the differentiation of fibroblasts into myofibroblasts. When the injury repair is repeated, the myofibroblasts proliferate excessively, forming fibrotic tissue. We goal to control myofibroblasts proliferation and apoptosis with anti-transforming growth factor-$\beta$ (anti-TGF-$\beta$) and anti-platelet-derived growth factor (anti-PDGF) medicines. The novel optimal regulator control problem with two controls (medicines) is proposed to simulate how to the preventing pulmonary fibrosis. Idiopathic pulmonary fibrosis (IPF) consists of restoring a system of cells, protein, and tissue networks with injury and scar. Myofibroblasts proliferation back to its equilibrium position after it has been disturbed by abnormal repair. Thus, the optimal regulator control problem with a parabolic partial differential equation as a constraint, zero flux boundary, and given specific initial conditions, is considered. The myofibroblast diffusion equation stands as a governing dynamic system while the objective function is the summation of myofibroblast, anti-TGF-$\beta$ and anti-PDGF medicines for the fixed final time. Here, myofibroblast is a nonlinear state of time while anti-TGF-$\beta$ and anti-PDGF are two unknown control functions. In order to solve the corresponding problem a weighted Galerkin method is used. Firstly, we convert the myofibroblast diffusion equation to a system of ordinary differential equations using the Lagrangian interpolation polynomials defined at Gauss-Lobatto integration points. Secondly, by the calculus of variations, the optimal control problem is solved successfully using canonical Hamiltonian and extended Riccati equations. Numerical results are given, and the plots are depicted. Moreover, solutions to the problem in which there is no control are compared. Numerical results show that, over time, the myofibroblast increases and then remains constant when there is no control. In contrast, the current solution decreases and vanishes after 300 days by prescribing controller medicines for anti-TGF-$\beta$ and anti-PDGF. The optimal strategy proposed in this paper helps practitioners to reduce myofibroblasts by controlling both anti-TGF-$\beta$ and anti-PDGF medicines.

## Introduction

After cell destruction, macrophages and other cells begin to produce inflammatory mediators (messenger molecules), including TGF-$\beta$ and PDGF to transform fibroblasts to myofibroblasts^1,2^. Myofibroblast cells appear during wound repair. Myofibroblasts secrete large amounts of extracellular matrix (ECM). The activity of these cells causes wound closure after injury. TGF-$\beta$ is a potent protein in enhancing collagen production by fibroblasts and myofibroblasts^3^. Moreover, PDGF proteins localized and sustained caused abnormal fibroblast proliferation and collagen production in IPF. There is PDGF protein production in macrophages and epithelial cells of patients but not in normal lung tissue^4^.

Hao first presented a mathematical model for sarcoidosis as a biomedical problem in 2014^5^, then by developing his model they used it for chronic pancreatitis^6^. A mathematical model of the interstitial fibrosis immune system is proposed by Hao et al.^7^ They monitored the effectiveness of existing anti-fibrotic drugs or those undergoing clinical trials in renal fibrosis. M1-derived inflammatory macrophages and M2 anti-inflammatory alveolar macrophages were considered for pulmonary fibrosis problems^8^. Hao et al. used this model to evaluate the effect of other potential drugs aimed at preventing liver fibrosis in 2017^9^.

Optimal control theory is a significant branch of modern control. Deals with the best possible control strategy that minimizes a certain performance index. It can be used as a powerful tool to solve the optimal control problem of disease dynamics. Solving optimization problems subject to constraints given in terms of partial differential equations (PDEs) is one of the most challenging problems. However, in medical, industrial, and economic applications, the transition from numerical simulations to optimal control problems is crucial. In order to overcome these difficulties model-based numerical simulation plays a central role. Many researchers have applied the optimal control problem to control the problems of cancer^10–12^ and infectious diseases^13–17^.

As an optimal control problem Mehrali-Varjani et al.^18^ solved a class of Hamilton Jacobi-bellman equations using pseudospectral methods in the year 2018. Abbasi and Malek^19^ presented hyperthermia cancer therapy by domain decomposition methods using strongly continuous semigroups in the year 2019. A pointwise optimal control solution for hyperthermia with thermal wave bioheat transfer was used by Abbasi and Malek^20^ in the year 2020. For the first time, in the year 2022, Bahram Yazdroudi and Malek^21^ proposed five model problems containing three optimal control problems and two dynamical systems for preventing the formation of pulmonary fibrosis by controlling TGF-$\beta$. They used approaches First Discretize, Then Optimize. For the discretization applied the central finite differences explicit method. They just control TGF-$\beta$. The differences between current research work and previous literature are:

1. No mathematical optimal control problem is solved for controlling the efficient parameters in fibrosis wounds^5,7–9^.
2. Just one control is used while the discretization is based on the central finite differences method in Bahram Yazdroudi and Malek^21^.
3. The system was not considered on there the time of inflammation and drug administration.

Here, some innovation approaches are:

(i) A novel dynamic system is modeled during the time of inflammation and drug administration.
(ii) A new hybrid optimal control problem with PDE constraint with two controls is applied.
(iii) An optimal control problem for decreasing myofibroblast is proposed where both anti-TGF-$\beta$ and anti-PDGF medicines are controlled by a novel technique.
(iv) The spectral method is used for discretization.
(v) The myofibroblast diffusion equation is converted to a system of ordinary differential equations using the Lagrangian interpolation polynomials defined at Gauss- Lobatto integration points.
(vi) Canonical Hamiltonian and extended Riccati equations for two controls are used.
(vii) the extended linear feedback is used to solve the related optimal control problem.
(viii) A constant vector *c* appears in the related state space ordinary differential equation (see Eq. 57).
(ix) The optimal strategy proposes to control both anti-TGF-$\beta$ and anti-PDGF medicines.

**Figure 1:**
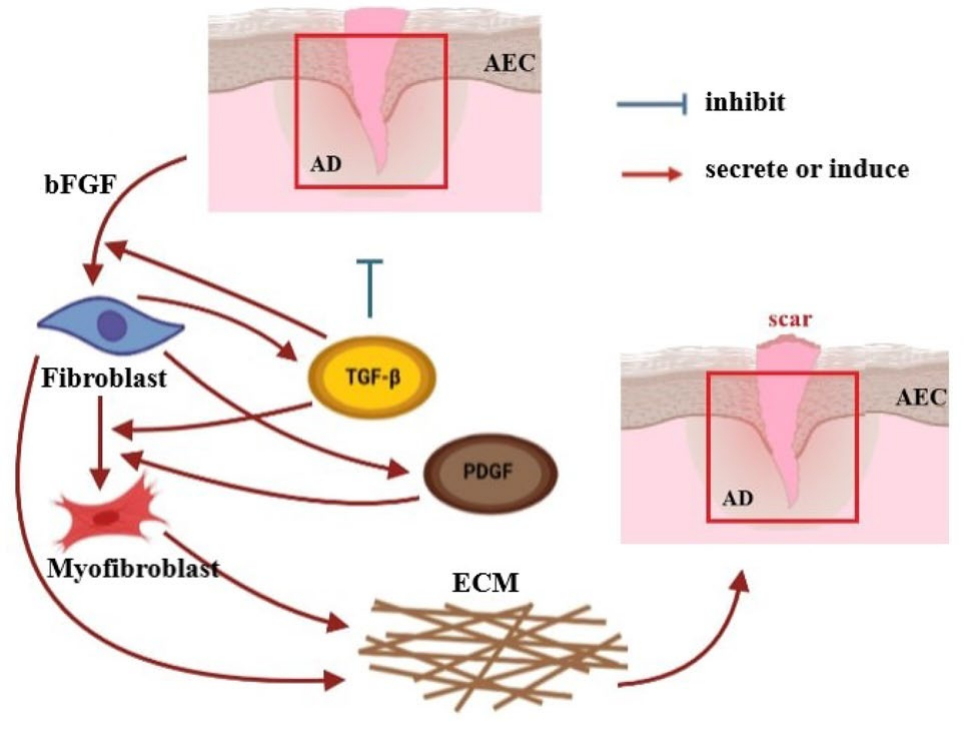
Part of schematic network for a cell, protein, and tissue in Idiopathic pulmonary fibrosis (IPF). When an injury occurs in an organ, the immune system secretes pro-inflammatory cytokines to suppress and respond to the injury. Inflammatory responses, if excessive, cause serious damage to the inflamed tissue^22^.

**Figure 2:**
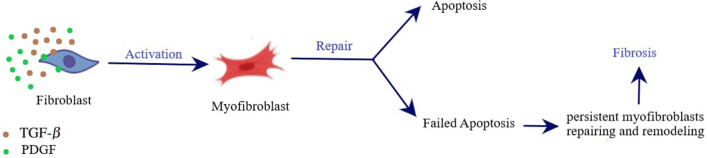
Differentiation of fibroblasts into myofibroblasts. Fibroblasts are activated and differentiate into myofibroblasts in response to tissue injury to initiate repair. Myofibroblasts secrete large amounts of ECM for repairing and remodeling. In normal repair, myofibroblasts vanish through apoptosis. In response to serious injury, myofibroblasts resist apoptosis. Their persistence leads to tissue remodeling and the formation of fibrosis.

**Figure 3:**
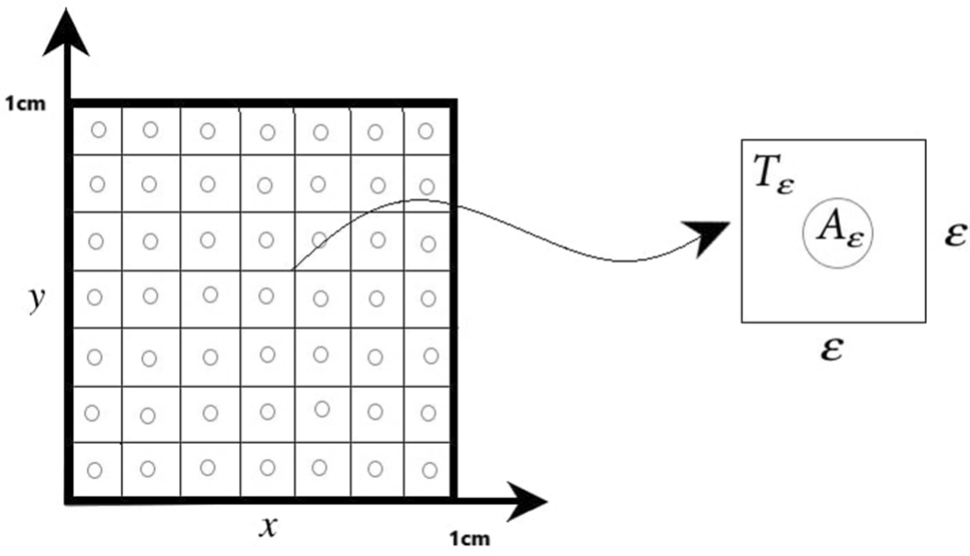
Lung geometry without damage area. Lung geometry consists of squares with smaller circles in the center that show the alveolar air space.

**Figure 4:**
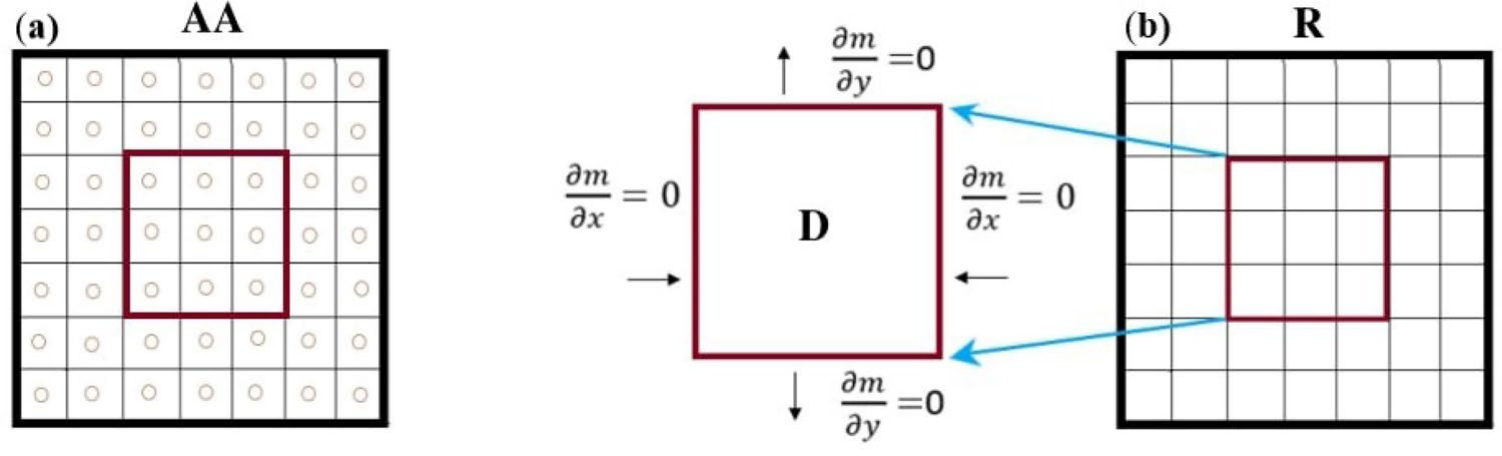
Lung geometry with damage area. In (**a**), the **AA** square is lung geometry consisting of lots of squares with small circles in the middle. Circles show the alveolar air space. In (**b**), the alveolar air space is not considered since circles are extremely small. A damaged area **D** is shown in the center of the square **R**. The boundary conditions for **D** have zero flux.

**Figure 5:**
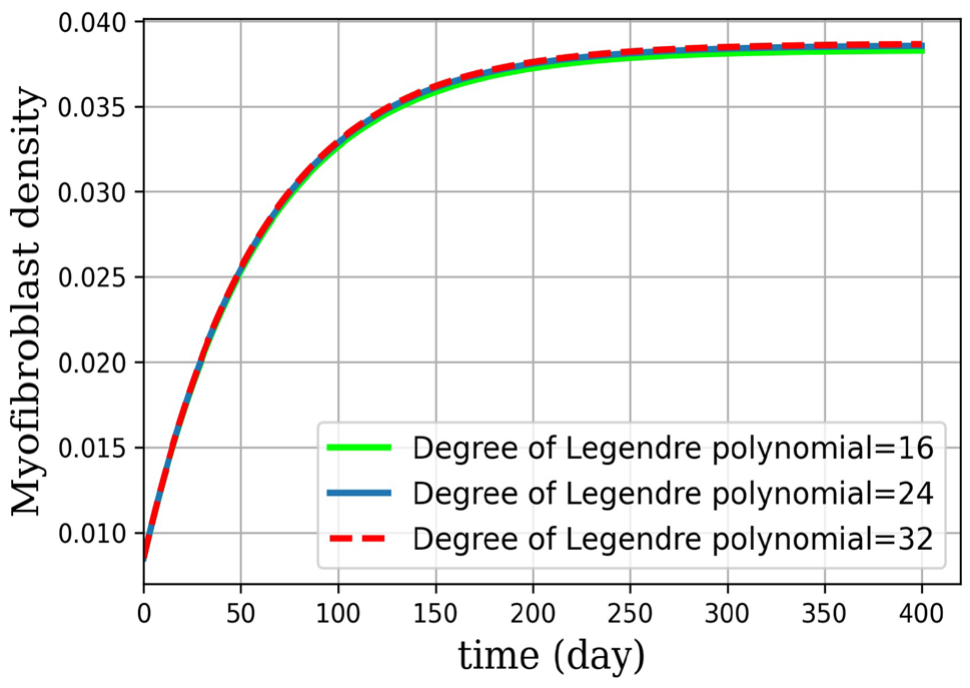
Dynamical system for myofibroblast density (no medication involves). The system of ODEs (35) are solved for $\hat{m}_N(t)$ during 400 days. Eq. (11) is discretized using the Lagrangian interpolation polynomials of order 16, 24, and 32 defined at Gauss-Lobatto integration points for green, blue, and red colors respectively. It is observed that, over time, the myofibroblast density increase to a certain amount. After approximately 300 days it remains constant but never vanishes. This shows that myofibroblasts resist apoptosis in response to serious injury. This means that if one uses no medication the persistent myofibroblast repairing leads to tissue remodeling and fibrosis formation^1,2^.

**Figure 6:**
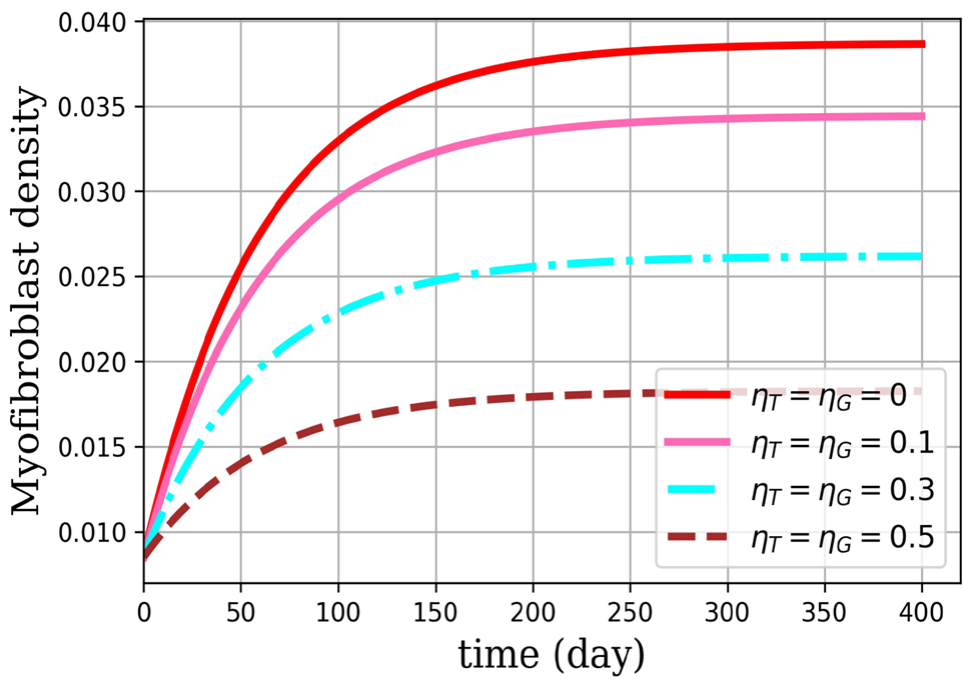
Dynamical system for myofibroblast density with different constant scalar values for $\eta _T(t)$ and $\eta _G(t)$ (two medicines are involved). Just Eq. (39) is solved for $\hat{m}(t)$ during 400 days. Eq. (36) is discretized using the Lagrangian spectrol method for $N=32$ with different $\eta _T(t)$ and $\eta _G(t)$ for $(\eta _T(t),\eta _G(t))=(0,0), (0.1,0.1), (0.3,0.3), (0.5,0.5)$. It is observed that, over time, the myofibroblast density increase to a certain value after approximately 300 days, and it remains constant. It is shown that although by increasing the rate of medications $\eta _T(t)$ and $\eta _G(t)$, myofibroblast density decreases, however, the patient is not going to be cured even if the medication is increased. In general myofibroblast density has an increasing form up to some days it will stay constant after and it never vanishes thus it never removes. This process shows that if one changes the medication doses self-willed, we cannot expect that the myofibroblast density will disappear and apoptosis will happen. This experience encouraged the authors to go for simulating the whole problem as an optimal control problem.

**Figure 7:**
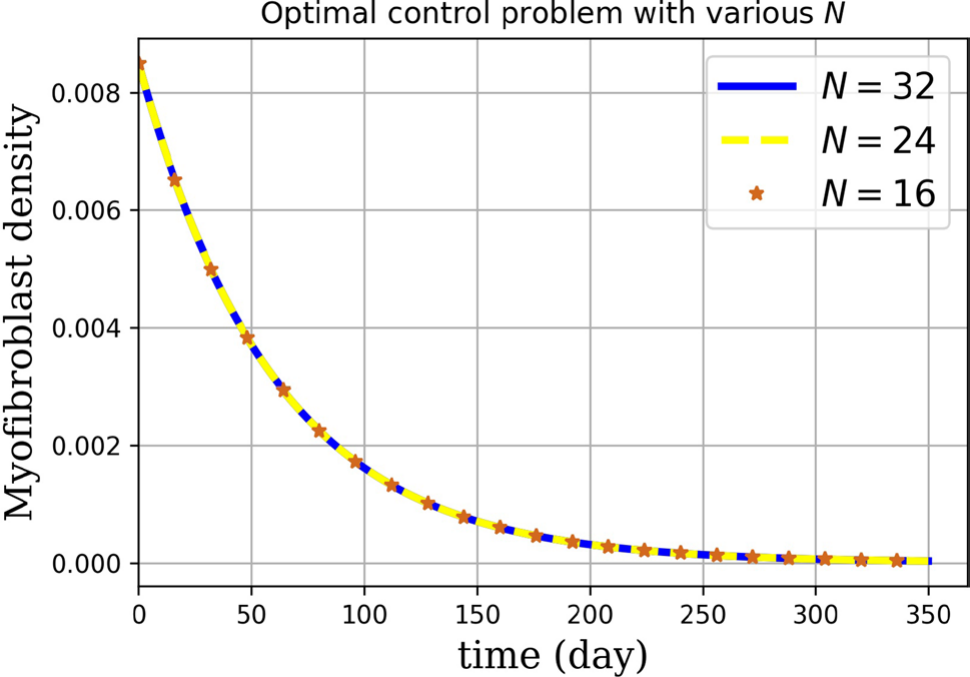
Novel optimal regulator control problem with different *N*.The optimal myofibroblast density with two controls is depicted. For solving Eqs. (41, 42) with initial and boundary conditions (6), we first convert Eq. (38) to a linear form (39). For discretization, we use the Lagrangian interpolation polynomials of orders 16, 24, and 32 defined at Gauss-Lobatto integration points. Then, the optimal control problem Eqs. [(41, 42) with initial and boundary conditions (6)] for different $N=16,24$, and 32 is solved. According to Table 3, the solution to the optimal control problem is more accurate for $N=32$.

**Figure 8:**
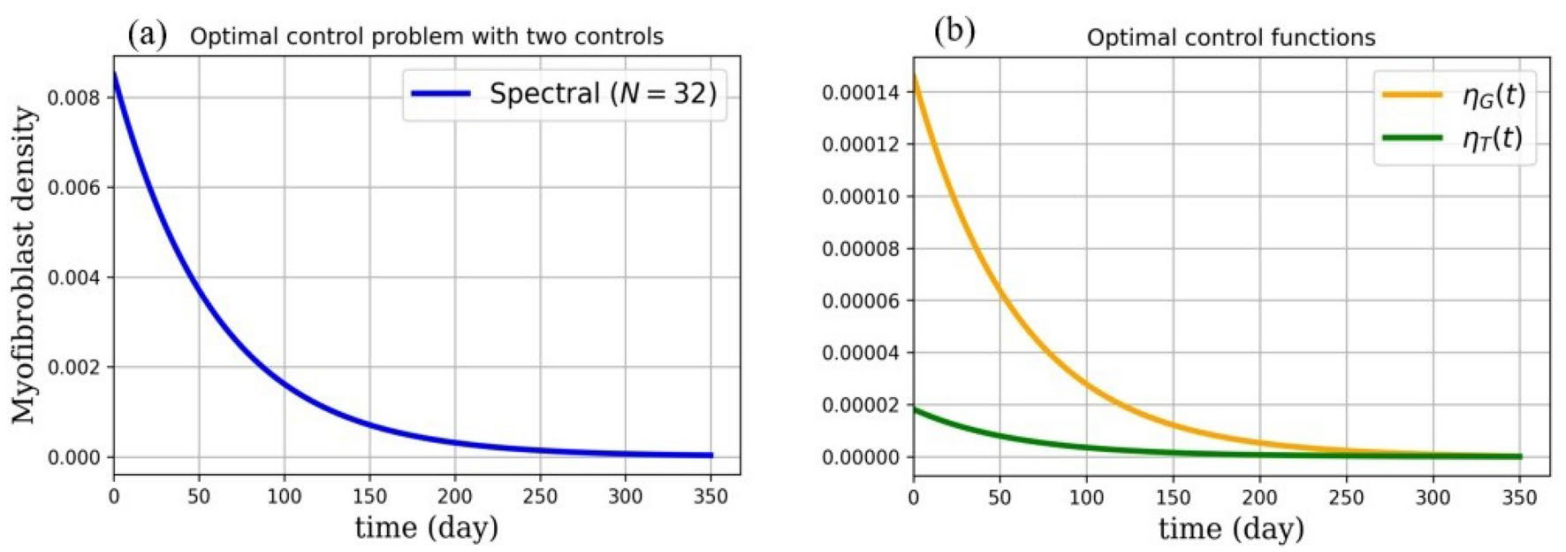
Novel optimal regulator control problem. In (**a**), optimal myofibroblast density with two controls is depicted using the spectral method ($N=32$). For solving Eqs. (41, 42) with initial and boundary conditions (6), we first transform Eq. (38) to a linear form (39). For discretization, we use the Lagrangian interpolation polynomials of order 32 defined at Gauss-Lobatto integration points. Then, we solve optimal control problem Eqs. (41, 42) with initial and boundary conditions (6) using Pontryagin’s minimum principle, Hamiltonian and developed Riccati equations. It is observed that when we control both anti-TGF-${\beta }$ and anti-PDGF the myofibroblast density vanishes after almost 300 days. This strategy can be applied by physicians when they prescribe anti-TGF-${\beta }$ and anti-PDGF medicines in almost 300 days. In (**b**), the optimal control functions $\eta _T(t)$ and $\eta _G(t)$ are depicted. It is observed that the control functions (anti-TGF-${\beta }$ and anti-PDGF) decrease and then remain zero. Hence, in repair tissue, myofibroblasts vanish through apoptosis, and no formation of fibrosis tissue happens. The medicines are prescribed in certain doses and decrease over time. With this strategy, there is no need to prescribe medicines during some last days of the patient take cure duration.

**Figure 9:**
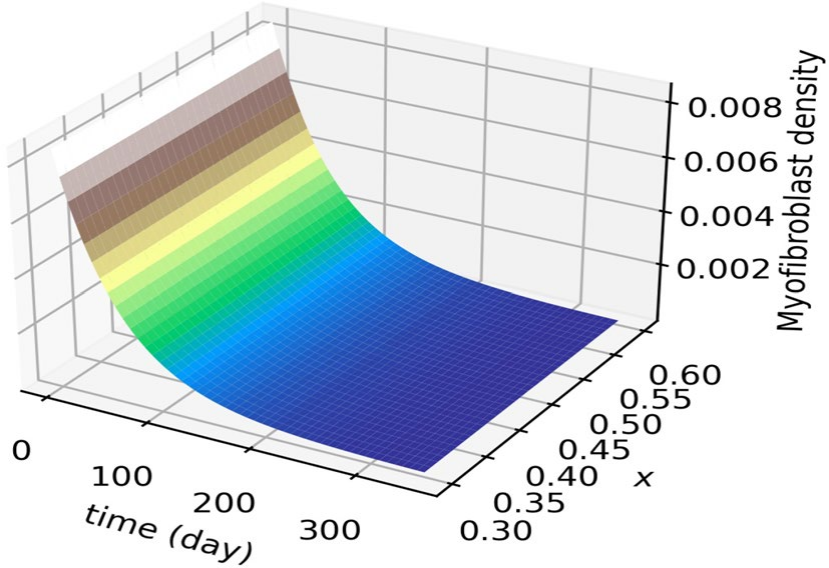
Novel optimal regulator control problem. Myofibroblast density against position (*x*) and time is plotted. It is observed that the myofibroblast density decreases and vanishes over time with controlling both $\eta _T(t)$ and $\eta _G(t)$.

**Figure 10:**
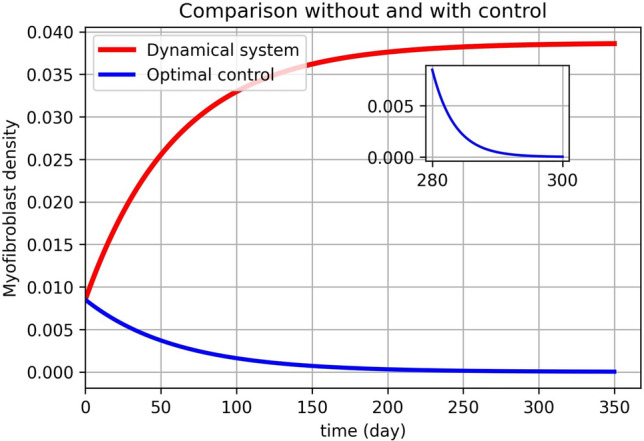
Comparison without and with control. In this figure, we compare two methods (without and with control) for myofibroblast density. A dynamical system is solved by spectral method $N=32$ (also see Fig. 5 red). It is observed that, over time, the myofibroblast density increase and then remains constant but never vanishes. The optimal regulator control problem is solved and optimal myofibroblast density is depicted by the blue line using the spectral method $N=32$ (also see Fig. 8a). It is observed that by controlling both TGF-${\beta }$ and anti-PDGF the myofibroblast density decreases and vanishes after almost 300 days.

**Figure 11:**
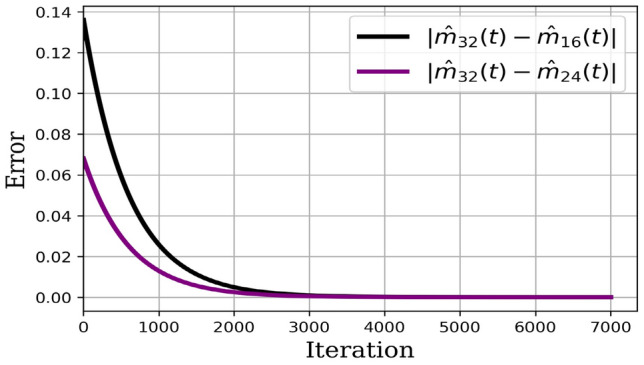
Absolute error dynamical system solutions. The absolute error for the solution of Eq. (35) with an approximation of the polynomial of degrees 32 and 24 is depicted by purple color. Moreover, the absolute error for the solution of Eq. (35) with an approximation of the polynomial of degrees 32 and 16 is depicted by black color.

**Figure 12:**
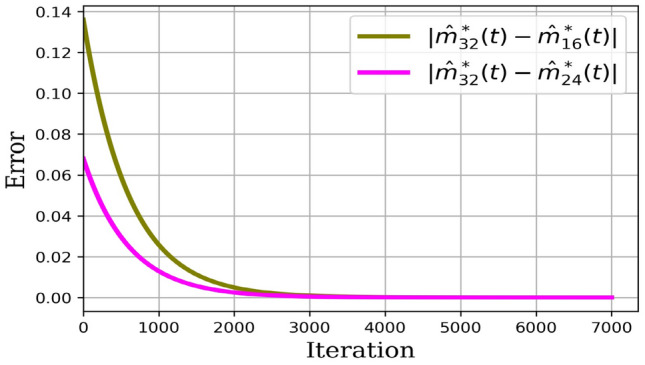
Absolute error optimal regulator control problem solutions. The absolute error for the solution of Eqs. [(41),(42), and (6)] using the spectral method with an approximation of the polynomial of degrees 32 and 24 is depicted in magenta color. Moreover, the absolute error for the solution of Eqs. [(41),(42), and (6)] using the spectral method with an approximation of the polynomial of degrees 32 and 16 is depicted in olive color.

In the present paper, in Fig. 1, IPF is shown schematically. In Fig. 2, differentiate myofibroblast from fibroblast is shown. In Figs. 3 and 4, lung tissue with and without damage area is shown. The myofibroblast diffusion and homogenized equations are proposed. The functions, variables, and parameters are given in Table 1. Legendre polynomials and Gauss-Lobatto integration points for Galerkin spectral method are introduced. This optimal control problem is solved by first discretizing and then optimizing technique. Firstly, the myofibroblast diffusion partial differential equation (PDE) is converted into an algebraic system of ordinary differential equations (ODEs) by the Galerkin spectral method. Secondly, the optimal control problem using Pontryagins minimum principle is solved. Numerical results are given in Figs. 5, 6, 7, 8, 9,10, 11, 12 and Tables 2, 3 and 4. Finally, discussion and conclusions are presented.

## Mathematical configuration

### Lung tissue simulation

Figure 1 shows part of the cell schematic network, proteins, and tissues in IPF.

We assume the lung tissue is a square with an edge size of 1 cm. The square is divided into small squares and is called $T_{\varepsilon }$ with an edge size of $\varepsilon$. A simple representation of the lung geometry with two dimensions of *x* and *y* is considered. In each small square, there is a concentric circle as alveolar air space ($A_{\varepsilon }$). Alveolar tissue is shown between the squares and circles in Fig. 3.

We assume that $\varepsilon$ is extremely small and close to zero then we face a homogenized alveolar tissue ($T_{\varepsilon }/ A_{\varepsilon }$). In this case, we ignore the alveoli space in the square and call it **R** square. Therefore, lung tissue is just a square without alveolar space, as shown in Fig. 4(b). Tissue inflammation is a small square **D** in **R** (**R** $= [0,1] \times [0,1]$). For a mild case of IPF, we assume that **D** $=0.3 \times 0.3$ $\hbox {cm}^{2}$^22^.

### Myofibroblast diffusion equation

According to the functions, variables, and parameters given in Table 1 the myofibroblast diffusion equation for one-dimension in $\mathbf { D_q}, q$ $=x$ or *y*. The myofibroblast diffusion equation for one-dimension is as follows:

1 $$\begin{aligned} \begin{aligned} \dfrac{ \partial m(x,t)}{ \partial t}- D_m {\nabla }^2 m(x,t)&=\underbrace{\Big (\lambda _{mfT} \dfrac{T_{GF}(x,t)}{K_{T_{GF}}+T_{GF}(x,t)}+\lambda _{mfG}\dfrac{G(x,t)}{K_G+G(x,t)}\Big )}_{f\rightarrow m} f-\underbrace{d_mm(x,t)}_{apoptosis}. \end{aligned} \end{aligned}$$

Where, $\nabla ^2 m(x,t) =\frac{\partial ^{2}m}{\partial x^2}$ and initial and boundary conditions are

2 $$\begin{aligned} \begin{aligned} {\left\{ \begin{array}{ll} m(x_0,t_0) = 8. 5 \times 10^{-3},\quad (x_0,t_0) =(0.3,0)\\ \dfrac{ \partial m(t)}{ \partial x}=0, \quad x = 0.3,0.6. \end{array}\right. } \end{aligned} \end{aligned}$$

The first term fibroblast transforms into myofibroblast by TGF-${\beta }$ and PDGF^23,24^. In Eq. (1), for simplicity, we set

3 $$\begin{aligned} c_m=\Big (\lambda _{mfT} \dfrac{T_{GF}(x,t)}{K_{T_{GF}}+T_{GF}(x,t)}+\lambda _{mfG}\dfrac{G(x,t)}{K_G+G(x,t)}\Big )f, \end{aligned}$$

thus, the dynamical equation myofibroblast diffusion with initial and boundary conditions (2) is as follows:

4 $$\begin{aligned} \dfrac{ \partial m(x,t)}{ \partial t}- D_m {\nabla }^2 m(x,t)=-d_mm(x,t)+c_m. \end{aligned}$$

The myofibroblast diffusion equation for two-dimension in damaged area ${\textbf {D}}$ is as follows:

5 $$\begin{aligned} \begin{aligned} \dfrac{ \partial m(x,y,t)}{ \partial t}- D_m {\nabla }^2 m(x,y,t)&=\Big (\lambda _{mfT} \dfrac{T_{GF}(x,y,t)}{K_{T_{GF}}+T_{GF}(x,y,t)}+\lambda _{mfG}\dfrac{G(x,y,t)}{K_G+G(x,y,t)}\Big ) f-d_mm(x,y,t), \end{aligned} \end{aligned}$$

where, $\nabla ^2 m(x,y,t) =\frac{\partial ^2m}{\partial x^2}+\frac{\partial ^2m}{\partial y^2}$, and initial and boundary conditions are

6 $$\begin{aligned} \begin{aligned} {\left\{ \begin{array}{ll} m(x_0,y_0,t_0) = 8. 5 \times 10^{-3},\quad (x_0,y_0,t_0) =(0.3,0.3,0)\\ \dfrac{ \partial m(t)}{ \partial x}=0, \quad x = 0.3,0.6,\\ \dfrac{\partial m(t)}{\partial y}=0, \quad y = 0.3,0.6. \end{array}\right. } \end{aligned} \end{aligned}$$

We set

7 $$\begin{aligned} c=\Big (\lambda _{mfT} \dfrac{T_{GF}(x,y,t)}{K_{T_{GF}}+T_{GF}(x,y,t)}+\lambda _{mfG}\dfrac{G(x,y,t)}{K_G+G(x,y,t)}\Big )f. \end{aligned}$$

**Table 1:** Nomenclator.

Parameter	Description	Value
$\eta _T(t)$	Anti-transforming growth factor-$\beta$ (anti-TGF-$\beta$)	
$\eta _G(t)$	Anti-platelet-derived growth factor (anti-PDGF)	
$T_{\varepsilon }$	Squares with smaller circles in the centers of each square in a unit square	
$T_{\varepsilon }/ A_{\varepsilon }$	Alveolar tissue	
*m*(*x*, *y*, *t*)	Myofibroblast density at (*x*, *y*) position, $t\in [0,350]$	
*G*(*x*, *y*, *t*)	Concentration of activated PDGF at (*x*, *y*) position, $t\in [0,350]$	
$T_{GF}(x,y,t)$	Concentration of activated TGF-$\beta$ at (*x*, *y*) position, $t\in [0,350]$	
$t_f$	Final time	
*a*	The homogenized coefficient for myofibroblast diffusion equation	0.11^8^
$\gamma$	The homogenized coefficient for myofibroblast diffusion equation	127/343^8^
$m(x_0,y_0,t_0)$	Initial value myofibroblast density	$8.5\times 10^{-3} \hbox {gcm}^{-3}$^7,8^, estimated
$G(x_0,y_0,t_0)$	Initial concentration of activated PDGF	$0.58\times 10^{-3} \hbox {gcm}^{-3}$^7,8^
$T_{GF}(x_0,y_0,t_0)$	Concentration of activated TGF-$\beta$	$2.51 \times 10^{-12} \hbox {gcm}^{-3}$^7,8^
*f*	Fibroblasts density in the region D are constant	$4.75\times 10^{-3} \hbox {gcm}^{-3}$^7,8^, estimated
$d_m$	Death rate of myofibroblasts	$1.66 \times 10^{-2} \hbox {day}^{-1}$^7,8^
$D_m$	The diffusion coefficient of myofibroblasts	$1.47 \times 10^{-5} \hbox {cm}^{2}day^{-1}$^7,8^
$\lambda _{mfT}$	Activation rate of myofibroblasts due to TGF-$\beta$	$1.2 \times 10^{-1} \hbox {day}^{-1}$^7,8^
$\lambda _{mfG}$	Activation rate of myofibroblast due to PDGF	$1.2 \times 10^{-1} \hbox {day}^{-1}$^7,8^
$K_G$	PDGF saturation for activation of myofibroblasts	$1.5 \times 10^{-8}$g/$\hbox {cm}^{-3}$^7,8^
$K_{T_{GF}}$	TGF-$\beta$ saturation for apoptosis for alveolar tissue apoptosis	$1 \times 10^{-10}$g/$\hbox {cm}^{-3}$^7,8^
**R**	The homogenized lung tissue	$[0,1]\times [0,1]$
**AA**	Alveolar tissue$+$Alveolar air space	$[0,1]\times [0,1]$
**D**	The homogenized damaged tissue in R	$[0.3,0.6]\times [0.3,0.6]$
$\mathbf {D_q}$	The homogenized damaged tissue in one-dimension, $\textbf{q}$ $=x$ or *y*	[0.3, 0.6]
**AD**	The damaged tissue in AA	$[0.3,0.6]\times [0.3,0.6]$
$A_{\varepsilon }$	Alveolar air space	extremely small

### The homogenized myofibroblast diffusion equation

According to Jikov et al.^25^ and Goel et al.^26^, the homogenized myofibroblast diffusion equation are

8 $$\begin{aligned} \gamma \dfrac{ \partial m(x,t)}{ \partial t}- D_m {\tilde{\nabla }}^2 m(x,t)=\gamma (-d_mm(x,t)+c_m) \quad \text { in} \quad \mathbf { D_q}, \end{aligned}$$

and

9 $$\begin{aligned} \gamma \dfrac{ \partial m(x,y,t)}{ \partial t}- D_m {\tilde{\nabla }}^2 m(x,y,t)=\gamma (-d_mm(x,y,t)+c) \quad \text { in} \quad \textbf{ D}, \end{aligned}$$

where $\gamma ={\frac{127}{343}}$, $\tilde{\nabla }^2= a\frac{\partial ^2}{\partial x^2}$, and $a=0.11$. We divide both sides of Eqs. (8) and (9) by $\gamma$. The homogenized myofibroblast diffusion equation for one and two dimensions are as follows

10 $$\begin{aligned} \dfrac{ \partial m(x,t) }{ \partial t}-r { \nabla }^2 m(x,t)=-d_mm(x,t)+c_m \quad r=\dfrac{aD_m}{\gamma }\qquad \text { in} \quad \mathbf { D_q}. \end{aligned}$$

and

11 $$\begin{aligned} \dfrac{\partial m(x,y,t)}{\partial t}-r {\nabla }^2 m(x,y,t)=-d_mm(x,y,t)+c \quad r=\dfrac{aD_m}{\gamma } \qquad \text { in} \quad \textbf{ D}. \end{aligned}$$

### Spectral method

This part of the paper includes the essential formulas for Legendre polynomials, and Legendre spectral method in one and two dimensions, together with the discretization technique^27–30^

### Legendre polynomials

The Legendre polynomials $L_k(\xi ), k = 0, 1,\ldots ,$ are the eigenfunctions of the singular Sturm-Liouville problem^27^:

12 $$\begin{aligned} ( (1-\xi ^2)L^{\prime }_k(\xi ) )^{\prime }+ k(k+1)L_k(\xi )=0. \end{aligned}$$

$L_k(\xi )$ is even when *k* is even and odd when *k* is odd. If $L_k(\xi )$ is normalized so that $L_k(1)= 1$. For each *k*, we get:

13 $$\begin{aligned} L_k(\xi )=\dfrac{1}{2^k}\sum ^{[k/2]}_{j=0} (-1)^j \genfrac(){0.0pt}0{k}{j}\genfrac(){0.0pt}0{2k-2j}{k}\xi ^{k-2j}, \end{aligned}$$

where [*k*/2] denotes the integral part of *k*/2. $L_0(\varvec{\xi }) = 1$ and $L_1(\varvec{\xi }) = \varvec{\xi }$. For each pair of Legendre polynomials of degrees *k* and *M*, the following orthogonality property applies

14 $$\begin{aligned} \int ^ 1_{-1} L_k(\xi )L_M(\xi )d\xi =\dfrac{2}{2k+1}\delta _{kM}, \end{aligned}$$

where $\delta _{kM}$ is Kronecker’s delta. The kth-degree Lobatto polynomial, $L0_k$, derives from the $(k + 1)$-degree Legendre polynomial, $L_{k+1}$, as

15 $$\begin{aligned} L0_k(\varvec{\xi })=L^{\prime }_{\varvec{k}+1}(\varvec{\xi }). \end{aligned}$$

Legendre and Lobatto polynomials can be calculated by the recursive relations^27^

16 $$\begin{aligned} L_{k+1}(\varvec{\xi })&=\dfrac{2k+1}{k+1}\varvec{\xi } L_k(\varvec{\xi })-\dfrac{k}{k+1}L_{k-1}(\varvec{\xi }), \end{aligned}$$

17 $$\begin{aligned} L0_{k-1}(\varvec{\xi })&=\dfrac{k(k+1)}{2k+1}\dfrac{(L_{k-1}(\varvec{\xi })-L_{k+1}(\varvec{\xi }))}{1-\varvec{\xi }^2}. \end{aligned}$$

### Legendre spectral method in one-dimension

Basis functions are the Lagrangian interpolation polynomials defined at Gauss-Lobatto integration points. We define the approximate the order $N=16,24,32$ for myofibroblast $m_N(x,t)$, as follows

18 $$\begin{aligned} m_N(x,t)=\sum ^N_{j=0} \hat{m}_j(x,t)\phi _j(x), \end{aligned}$$

where, $\hat{m}_j(x,t)$ is the discrete polynomial coefficient of $m_j(x,t)$ and $\phi _j$ is the *j* th Lagrange polynomial of order *N* on the Gauss-Legendre-Lobatto (GLL) points $\{ \xi _j \}^N_{j=0}$ and

19 $$\begin{aligned} \phi _j(\varvec{\xi })=\dfrac{1}{N(N+1)L_N(\varvec{\xi }_j)}\dfrac{(\varvec{\xi }^2-1)L^{\prime }_N(\varvec{\xi })}{\varvec{\xi }-\varvec{\xi }_j}\quad \quad 0\le j \le N, \quad -1\le \varvec{\xi } \le 1,\quad \varvec{\xi } \ne \varvec{\xi }_j, \end{aligned}$$

in which $L_N$ and $L^{\prime }_N$ are the Legendre polynomial of order *N* and its derivative, respectively. To convert the $[-1, 1]$ to [*a*, *b*], we use the mapping function while inverse mapping yields.

$$\begin{aligned} x(\varvec{\xi })&=\dfrac{(x_b-x_a)\varvec{\xi }}{2}+\dfrac{x_b+x_a}{2}\quad -1\le \varvec{\xi }\le 1, \\ \varvec{\xi }(x)&=\dfrac{2x-(x_b+x_a)}{x_b-x_a}\quad \quad x_a\le x\le x_b. \end{aligned}$$

For $h=x_b-x_a$, the stiffness^28^ ($S_q$), mass ($K_q$) and constant coefficients ($C_q$) matrices are as follows and $q=x,y$:

20 $$\begin{aligned} {S_q}_{i,j}&=\int ^{x_b}_{x_a}\phi ^{\prime }_i(x) \phi ^{\prime }_j(x)dx=\dfrac{2}{h}\int ^ 1_{-1}\phi ^{\prime }_i(\varvec{\xi }) \phi ^{\prime }_j(\varvec{\xi })d\varvec{\xi }, \end{aligned}$$

21 $$\begin{aligned} {K_q}_{i,j}&=\int ^{x_b}_{x_a}\phi _i(x) \phi _j(x)dx=\dfrac{h}{2}\int ^ 1_{-1}\phi _i(\varvec{\xi }) \phi _j(\varvec{\xi })d\varvec{\xi }, \end{aligned}$$

22 $$\begin{aligned} {C_q}_{i,j}&=\int ^{x_b}_{x_a}\phi _i(x)d\varvec{\xi }. \end{aligned}$$

Using the Gauss quadrature, we have^27^

23 $$\begin{aligned} {S_q}_{ij}&=\dfrac{2}{h}\sum ^ N_{k=0} d_{ik}d_{jk}w_k, \end{aligned}$$

24 $$\begin{aligned} {K_q}_{ij}&=\dfrac{h}{2}\delta _{ij}w_i, \end{aligned}$$

where the GLL quadrature weights $\{w_k\}^N_{k=1}$ are given in the following

25 $$\begin{aligned} w_k&=\dfrac{2}{N(N+1)[L_N(\varvec{\xi })]^2} \quad 0\le k \le N, \end{aligned}$$

26 $$\begin{aligned} d_{ij}&= {\left\{ \begin{array}{ll} \dfrac{-N(N+1)}{4} &{}\quad i=j=0\\ 0 &{}\quad i=j\in [1,N-1] \\ \\ \dfrac{N(N+1)}{4}&{}\quad i=j=N\\ \\ \dfrac{L_N(\varvec{\xi }_i)}{L_N(\varvec{\xi }_j)}\dfrac{1}{\varvec{\xi }_ i -\varvec{\xi }_ j}&{}\quad i\ne j \end{array}\right. } \end{aligned}$$

The mass matrix is diagonal when the nodal points are the same as the quadrature points since the Lagrange polynomials have cardinality properties^29,30^.

For $q=x$ or *y*, we define $H^1 (D_{q})$ and $H^1 _0(D_{q})$ spaces as follows:

27 $$\begin{aligned} H^1 (D_{q})= \{v \in D_{q}, \quad \dfrac{\partial v}{\partial q}\in D_{q} \}, \quad H^1 _0(D_{q})=\{v \in H^1(D_{q}), \quad v|_{ \partial D_{q} }=0 \} \end{aligned}$$

For Eq. (10), proper approximation for $m_N(x,t)$ applies as a weighted Galerkin method. Find $\hat{m}_N(q,t) \in H^1 _0 (D_{q})$ such that for all $\phi \in H^1 _0(D_{q})$.

28 $$\begin{aligned} \int _{D_{x}} \phi _i\big (\dfrac{ \partial m_N(x,t)}{ \partial t}- \dfrac{ \partial }{\partial x}(r \dfrac{ \partial m_N(x,t) }{\partial x})+d_mm_N(x,t)-c_m\big )dD_{x}=0. \end{aligned}$$

We apply the Green theory and get the weak form as follows

29 $$\begin{aligned}{}&\int \phi _i\dfrac{ \partial m_N(x,t)}{\partial t}dx-\int _{\partial D_{x}}\phi _i \dfrac{ \partial m_N(x,t)}{\partial x}dx-\int r\dfrac{\partial \phi _i}{\partial x}\dfrac{\partial m_N(x,t)}{\partial x}dx+d_m\int \phi _i m_N(x,t)dx-c_m\int \phi _i dx=0. \end{aligned}$$

From the boundary condition $(\dfrac{ \partial m_N(x,t)}{\partial x}=0)$,

30 $$\begin{aligned} \int \phi _i\dfrac{ \partial m_N(x,t)}{\partial t}dx-\int r\dfrac{ \partial \phi _i}{\partial x}\dfrac{ \partial m_N(x,t)}{\partial x}dx+d_m\int \phi _i m_N(x,t)dx-c_m\int \phi _i dx=0. \end{aligned}$$

We substitute (18) in (30). Thus, $\hat{m}_N(x,t)$ can be determined by solving the following ODE systems where the entries of the $c_m$, $S_x$, $K_x$, and $C_x$ are defined in (3), (20), (21), and (22).

31 $$\begin{aligned}{}&K_x\dot{\hat{m}}_N(x,t)(t)-rS_x\hat{m}_N(x,t)+d_mK_x\hat{m}_N(x,t)-c_mC_x=0, \end{aligned}$$

32 $$\begin{aligned}{}&\dot{\hat{m}}_N(x,t)=K_x^{-1}((-d_mK_x+rS_x)\hat{m}_N(x,t)+c_mC_x). \end{aligned}$$

### Legendre spectral method in two-dimensions

We assume that the domain considered is partitioned into the quadrilateral where $[-1,1] \times [-1,1]$ is the reference square. The local approximating functions are the tensor product of the one-dimensional Legendre polynomials. The approximation of order *N* for the unknown function $m_N(x,y,t)$ in the reference square is as follows:

33 $$\begin{aligned} m_N(x,y,t)=\sum ^N_{j=0} \hat{m}_j(x,y,t)\phi _ j(x) \phi _ j(y). \end{aligned}$$

The stiffness matrix *S*, the mass matrix *K*, and constant coefficients *C* matrices^28^, respectively, are defined as:

34 $$\begin{aligned} \begin{aligned} S&=S_x\otimes S_y,\\ K&=K_x\otimes K_y,\\ C&=C_x\otimes C_y, \end{aligned} \end{aligned}$$

where the entries of the $S_x$ and $S_y$ are defined in (20), the entries of the $K_x$ and $K_y$ are defined in (21), and the entries of the $C_x$ and $C_y$ are defined in (22). For (11), a suitable approximation for $m_N(x,y,t)$ applies as a weighted Galerkin method thus

35 $$\begin{aligned} \dot{\hat{m}}_N(x,y,t)=K^{-1}((-d_mK+rS)\hat{m}_N(x,y,t)+cC). \end{aligned}$$

For simplicity, we use the notion $\hat{m}_N(x,y,t) = \hat{m}(t)$ for $N=32$.

### The homogenized myofibroblast diffusion with medicines dynamical system

After cell destruction and using medicines, the myofibroblast diffusion Eq. is (11) changed to

36 $$\begin{aligned} \dfrac{ \partial m(x,y,t)}{ \partial t}- r {\nabla }^2 m(x,y,t)&= \lambda _{mfT} \dfrac{T_{GF}(x,y,t)f}{K_{T_{GF}}+T_{GF}(x,y,t)} (1-\eta _T(t))+\lambda _{mfG}\dfrac{G(x,y,t)f}{K_G+G(x,y,t)} (1-\eta _G(t))-d_mm(x,y,t), \end{aligned}$$

where $\eta _T(t)$ is anti-TGF-$\beta$ and $\eta _G(t)$ is anti-PDGF. It is clear that if $\eta _T(t)=\eta _G(t)=0$ then Eq. (36) is equal to Eq. (11).

### Optimal regulator control problem

The homogenized myofibroblast diffusion Eq. (36) using the initial and boundary conditions (6) stands as a governing dynamic system while the objective function is the summation of myofibroblast, anti-TGF-$\beta$ and anti-PDGF medicines for the fixed final time. From now on, for simplicity, we use the following notions $m(x,y,t) = m(t), T_{GF}(x,y,t) = T_{GF}(t)$ and $G(x,y,t) = G(t)$.

37 $$\begin{aligned} \min _{m(t),\eta _T(t),\eta _G(t)} J(m(t),\eta _T(t),\eta _G(t),t)&=\dfrac{1}{2}\int _{t_0}^{t_f}m(t)^2 dt+\dfrac{1}{2}\int _{t_0}^{t_f}\left( \eta _T(t)^2+\eta _G(t)^2\right) dt, \end{aligned}$$

38 $$\begin{aligned}{}&s.t. \nonumber \\ \dfrac{ \partial m(t)}{ \partial t}-r{\nabla }^2 m(t)&=-\lambda _{mfT}\dfrac{T_{GF}(t)f}{K_{T_{GF}}+T_{GF}(t)}\eta _T(t)-\lambda _{mfG}\dfrac{G(t)f}{K_G+G(t)}\eta _G (t)+c -d_mm(t), \end{aligned}$$

where *c* is defined in Eq. (7). $J(m(t),\eta _T(t),\eta _G(t),t): \mathbb {R}^2\times \mathbb {R}^2 \rightarrow \mathbb {R}$ is the objective functional consists of two terms $m(t): \mathbb {R}^2\rightarrow \mathbb {R}$ (is the state function), $(\eta _T(t),\eta _G(t)): \mathbb {R}^2\rightarrow \mathbb {R}$ (is the control function). $r=\dfrac{aD_m}{\gamma }$ is a parmeter, $\lambda _{mfT}$ is the activation rate of myofibroblast due to TGF-$\beta$, and $\lambda _{mfG}$ is activation rate of myofibroblast due to PDGF, $d_m$ is the death rate of myofibroblasts, $K_G$ is PDGF saturation for activation of myofibroblasts, $K_{T_{GF}}$ is TGF-$\beta$ saturation for apoptosis for alveolar tissue apoptosis, *f* is fibroblasts density, $T_{GF}(t)$ is the concentration of activated TGF-$\beta$ at (*x*, *y*) position, and *G* is the concentration of activated PDGF at (*x*, *y*) position, $t \in [0,350]$. (the value of parameters are described in see Table 1). Note that Eq. (38) is a system that considers the time of inflammation and drug administration while Eq. (11) is not. Legendre spectral method is used to discretize Eq. (38). Thus we deal with the following ODEs (see Legendre spectral method).

39 $$\begin{aligned} \begin{aligned} \dot{\hat{m}}(t)=A\hat{m}(t)+B_T\eta _T(t)+B_G\eta _G(t)+C_b, \end{aligned} \end{aligned}$$

where using (22), (34), and (35), we get *A*, $B_T$, $B_G$, and $C_b$ as follows

40 $$\begin{aligned} \begin{aligned} A=K^{-1}(-d_mK+rS),\quad B_T=-K^{-1}\int ^{x_b}_{x_a}\lambda _{mfT}\dfrac{T_{GF}(t)f}{K_{T_{GF}}+T_{GF}(t)}\phi _i,\quad B_G=-K^{-1}\int ^{x_b}_{x_a} \lambda _{mfG}\dfrac{G(t)f}{K_{G}+G(t)}\phi _i,\quad C_b=cK^{-1}C. \end{aligned} \end{aligned}$$

Thus, the discrete optimal control problem is

41 $$\begin{aligned} \min _{\hat{m}(t), \eta _T(t) ,\eta _G(t)} J(\hat{m}(t),\eta _T(t) ,\eta _G(t),t)&=\dfrac{1}{2}\int _{t_0}^{t_f}\hat{m}(t)^2dt+\dfrac{1}{2}\int _{t_0}^{t_f}(\eta _T(t)^2+\eta _T(t)^2) dt, \end{aligned}$$

42 $$\begin{aligned}{}&s.t. \nonumber \\ \dot{\hat{m}}(t)&=A\hat{m}(t)+B_T\eta _T(t)+B_G\eta _G(t)+C_b. \end{aligned}$$

In the next, we apply Pontryagin’s minimum principle^31,32^.

### Pontryagin’s minimum principle

The minimization of the performance index *J* will be done using Pontryagin’s minimum principle. The extended Hamiltonian for (41) and (42) is

43 $$\begin{aligned} \begin{aligned}{}&\tilde{H}(\varvec{\hat{m}}(t),\varvec{\eta _T}(t),\varvec{\eta _G}(t),\varvec{\lambda }(t),t)=\varvec{\hat{m}}(t)^2+ \varvec{\eta _T}(t)^2+\varvec{\eta _G}(t)^2+ \varvec{\lambda }^T(t)[A\varvec{\hat{m}}(t)+B_T\varvec{\eta _T}(t)+B_G\varvec{\eta _G}(t)+C_b]), \end{aligned} \end{aligned}$$

where, $\varvec{\lambda }(t)$ is the vector of the Lagrange multipliers. Define $\tilde{J}$ by

44 $$\begin{aligned} \begin{aligned} \tilde{J}&= \int _{t_0}^{t_{f}}[\tilde{H}(\varvec{\hat{m}}(t),\varvec{\eta _T}(t),\varvec{\eta _G}(t),\varvec{\lambda }(t),t)-\varvec{\lambda }^{T}(t) \varvec{\dot{\hat{m}}}(t)]dt. \end{aligned} \end{aligned}$$

The first differential $\tilde{J}$ with respect to the vectors $\hat{m}(t)$, $\eta _T(t)$ and $\eta _G(t)$ are given by

45 $$\begin{aligned} \begin{aligned} \delta {\tilde{J}}= \int _{t_0}^{t_{f}}(\delta \hat{m}^T[\dfrac{\partial H}{\partial \hat{m}}+\dot{\lambda }(t)]+\delta \eta _T^T[\dfrac{\partial H}{\partial \eta _T}]+\delta \eta _G^T[\dfrac{\partial H}{\partial \eta _G}])dt. \end{aligned} \end{aligned}$$

A necessary condition for the performance index $\tilde{J}$ to a minimum is that $\delta {\tilde{J}}=0$. Thus, the vectors $\varvec{\hat{m}}(t)$, $\eta _T(t)$ and $\eta _G(t)$ must satisfy in the following equations

46 $$\begin{aligned}{}&\dfrac{\partial \tilde{H}(\varvec{\hat{m}^*}(t),\varvec{\eta _T^*}(t),\varvec{\eta _G^*}(t),\varvec{\lambda ^*}(t),t)}{\partial \varvec{\hat{m}}}=-\varvec{\dot{\lambda }^*}(t), \end{aligned}$$

47 $$\begin{aligned}{}&\dfrac{\partial \tilde{H}(\varvec{\hat{m}^*}(t),\varvec{\eta _T^*}(t),\varvec{\eta _G^*}(t),\varvec{\lambda ^*}(t),t)}{\partial \varvec{\lambda }}=\varvec{\dot{\hat{m}}^*}(t), \end{aligned}$$

48 $$\begin{aligned}{}&\tilde{H}(\varvec{\hat{m}^*}(t),\varvec{\eta _T^*}(t),\varvec{\eta _G^*}(t),\varvec{\lambda ^*}(t),t)\le \tilde{H}(\varvec{\hat{m}^*}(t),\varvec{\eta _T}(t),\varvec{\eta _G^*}(t),\varvec{\lambda ^*}(t),t) \end{aligned}$$

49 $$\begin{aligned}{}&\tilde{H}(\varvec{\hat{m}^*}(t),\varvec{\eta _T^*}(t),\varvec{\eta _G^*}(t),\varvec{\lambda ^*}(t),t)\le \tilde{H}(\varvec{\hat{m}^*}(t),\varvec{\eta _T^*}(t),\varvec{\eta _G}(t),\varvec{\lambda ^*}(t),t). \end{aligned}$$

In this case, for $\eta _T^{*}(t)$ and $\eta _G^{*}(t)$ to minimize the Hamiltonian equation, it is necessary that

50 $$\begin{aligned} \dfrac{\partial \tilde{H}(\varvec{\hat{m}^*}(t),\varvec{\eta _T^*}(t),\varvec{\eta _G^*}(t),\varvec{\lambda ^*}(t),t)}{\partial \varvec{\eta _T}}&=0, \end{aligned}$$

51 $$\begin{aligned} \dfrac{\partial \tilde{H}(\varvec{\hat{m}^*}(t),\varvec{\eta _T^*}(t),\varvec{\eta _G^*}(t),\varvec{\lambda ^*}(t),t)}{\partial \varvec{\eta _G}}&=0. \end{aligned}$$

If Eqs. (50) and (51) are satisfied, and matrices $\dfrac{\partial ^2 \tilde{H}(\varvec{\hat{m}^*}(t),\varvec{\eta _T^*}(t),\varvec{\eta _G^*}(t),\varvec{\lambda ^*}(t),t)}{\partial \varvec{\eta _T}^2 }$ and $\dfrac{\partial ^2 \tilde{H}(\varvec{\hat{m}^*}(t),\varvec{\eta _T^*}(t),\varvec{\eta _G^*}(t),\varvec{\lambda ^*}(t),t)}{\partial \varvec{\eta _G}^2 }$ are positive definite, this is sufficient to guarantee that $\varvec{\eta _T^*}(t)$ and $\varvec{\eta _G^*}(t)$ causes $\tilde{H}(\varvec{\hat{m}}(t),\varvec{\eta _T}(t),\varvec{\eta _G}(t),\varvec{\lambda }(t),t)$ to be a local minimum.

52 $$\begin{aligned} \begin{aligned} \varvec{\eta _T^*}(t)&=-B_T^{T}\varvec{\lambda ^*}(t),\\ \varvec{\eta _G^*}(t)&=-B_G^{T}\varvec{\lambda ^*}(t). \end{aligned} \end{aligned}$$

We use the linear feedback form for finding the optimal control, that is, look for functions $K_T(t)$ and $K_g(t)$.

53 $$\begin{aligned} \begin{aligned} \varvec{\eta ^*_T}(t)&=K_T(t)\varvec{\hat{m}^*}(t)+\rho _T,\\ \varvec{\eta ^*_G}(t)&=K_g(t)\varvec{\hat{m}^*}(t)+\rho _G. \end{aligned} \end{aligned}$$

For the unknowns $\rho _T$, $\rho _G$, $K_T(t)$ and $K_g(t)$ as the feedback matrices, we assume that the vector of the Lagrange multiplier $\varvec{\lambda ^*}(t)$ is linear in $\varvec{\hat{m}^*}(t)$, i.e.

54 $$\begin{aligned} \varvec{\lambda ^*}(t)=\varvec{p}(t)\varvec{\hat{m}^*}(t)+cg(t) \end{aligned}$$

for the unknown $\varvec{p}(t)$ and *g*(*t*) if we substitute Eq. (54) in Eq. (52), we have

55 $$\begin{aligned} \begin{aligned} \varvec{\eta ^*_T}(t)&=-B_T^{T}(\varvec{p}(t)\varvec{\hat{m}^*}(t)+cg(t)),\\ \varvec{\eta ^*_G}(t)&=-B_G^{T}(\varvec{p}(t)\varvec{\hat{m}^*}(t)+cg(t)). \end{aligned} \end{aligned}$$

By comparing (53) and (55), we have

56 $$\begin{aligned} \begin{aligned} K_T(t)=-B_T^{T}\varvec{p}(t), \quad K_g(t)=-B_G^{T}\varvec{p}(t), \quad \rho _T= -cB_Tg(t),\quad \rho _G= -cB_Gg(t). \end{aligned} \end{aligned}$$

By substitute Eq. (55) in Eq. (42), we have

57 $$\begin{aligned} \begin{aligned} \varvec{\dot{\hat{m}}}^*(t)=A\varvec{\hat{m}}^*(t)+B_T(-B_T^{T}\varvec{p}(t)\varvec{\hat{m}^*(t)}-B_T^{T}cg(t))+B_G(-B_G^{T}\varvec{p}(t)\varvec{\hat{m}}^*(t)-cB_G^{T}g(t))+c. \end{aligned} \end{aligned}$$

From differentiate (54) and using (46), we have

58 $$\begin{aligned} \begin{aligned} \varvec{\dot{\lambda }}^*(t)=\dot{p}(t)\hat{m}^*(t)+p(t)\dot{\hat{m}}^*(t)+c\dot{g}(t)=-\varvec{\hat{m}^*}(t) - A^{T}\varvec{\lambda ^*}(t). \end{aligned} \end{aligned}$$

Finally, if we subsitute (54) and (57) in Eq. (58)

59 $$\begin{aligned} \begin{aligned}{}&\dot{p}(t)\hat{m}^*(t)+p(t)A\hat{m}^*(t)-p(t)B_Tp(t)\hat{m}^*(t)B_T-p(t)B_Gp(t)\hat{m}^*(t)B_G-cp(t)B_Tg(t)B_T-p(t)B_Gg(t)B_Gc+cp(t)\\&+c\dot{g}(t)= -\hat{m}^*(t)-Ap(t)\hat{m}^*(t)-cAg(t) \end{aligned} \end{aligned}$$

We get

60 $$\begin{aligned} \begin{aligned}{}&\hat{m}^*(t)\big (\dot{p}(t)+p(t)A-p(t)B_Tp(t)B_T-p(t)B_Gp(t)B_G+I+Ap(t)\big )+c\big (-p(t)B_Tg(t)B_T-p(t)B_Gg(t)B_G+p(t)\\&+\dot{g}(t)+Ag(t)\big )=0 \end{aligned} \end{aligned}$$

In Eq. (60), $\hat{m}^*(t)$ and *c* are positive and not zero. Thus, the coefficient of $\hat{m}(t)$ and the second term must be equal to zero simultaneously. Therefore Eq. (60) reduces to two differential equations (developed Riccati equations) as follows

61 $$\begin{aligned} \begin{aligned}{}&\dot{p}(t)+p(t)A-p(t)B_Tp(t)B_T-p(t)B_Gp(t)B_G+I+AP(t)=0\\&\dot{g}(t)-p(t)B_Tg(t)B_T-p(t)B_Gg(t)B_G+p(t)+Ag(t)=0 \end{aligned} \end{aligned}$$

We solve (61) by the Euler approximation method and using (54). Firstly, we find *p*(*t*), *g*(*t*) and $\varvec{{\lambda }^*}(t)$ from (61 and 58). Secondly, the problem (41) and (42) with initial and boundary conditions (6) can be solve.

## Numerical results

Numerical results are done using Python programming software version 3.8, while the processor is AMD Ryzen 5 5500U.

Numerical results are presented as follows:

(i) * Just the dynamical system solution (no medication involves)* The dynamical system is solved by transforming the related PDE to a system of ODEs using Lagrangian interpolation polynomials of order 16, 24, and 32 defined at Gauss-Lobatto integration points. In Fig. 5, the dynamical system for myofibroblast density (11) with initial and boundary conditions (6) is solved. Myofibroblast density against time is plotted and compared.
(ii) *Just dynamical system solutions with different constant scalar values for* $\eta _T(t)$ *and* $\eta _G(t)$ *(two medicines are involved)* By keeping the functions $\eta _T(t)$ and $\eta _G(t)$ as constant scalar values
  $(\eta _T(t),\eta _G(t))=(0,0), (0.1,0.1), (0.3,0.3), (0.5,0.5)$ in the dynamical system (36) myofibroblast densities with different dosages of medications are computed and in Fig. 6 solutions are plotted. As it is shown in a problem simulation just with the dynamical system without medicines (11) and for the dynamical system with medicines (36) one can not cure the patient in this way. The reason is that the fibroblast density never vanishes and therefore never removes. Thus, to decrease and vanish myofibroblast density, one needs to change the problem formulation (36) to an optimal control problem. To do this, in the next steps the optimal regulator control problem (41, 42) with initial and boundary conditions (6) is proposed.
(iii) *Optimal regulator control problem solution (two medications as controls are involved)* In the existence of two controls (medications) using the First Discretize, Then Optimize technique the optimal control problem in (37, 38) is solved. In Fig. 7, the optimal control problem solutions for myofibroblast density with two controls [Eqs. (41, 42)), initial and boundary conditions (6)] with different *N* (16,24,32) is depicted. In Table 3, the optimal control problem solutions by the Lagrangian interpolation polynomials with 16, 24, and 32 degrees of freedom at times $t= 50, 100, 150, 250$ are shown. From this Table, one can recognize that even for moderate degrees of freedom solutions are accurate to 7 decimal points. From Table 4 one can find that $\hat{m}^*_{32}(t)$ gives a more accurate solution. Since, it is observed that the solution for $N=32$ is more efficient, thus from now on, we use $N=32$ in all of the computations. In Fig. 8(a), solution for optimal control problem Eqs. (41, 42) and (6) for myofibroblast density in point $x=y=0.45$ when two controls (anti-TGF-${\beta }$ and anti-PDGF) appear in the related dynamical system using the spectral method are depicted. Fig. 8(b) shows behavior of anti-TGF-${\beta }$ and anti-PDGF in the center of the regian **D** during 350 days. In Fig. 9, the behavior of myofibroblast density against position (*x*) and time is plotted. It is observed that the myofibroblast density in the existence of both $\eta _T(t)$ and $\eta _G(t)$ decreases and vanishes after 300 days.
(iv) *Comparison between the solutions without and with two controls* Fig. 10 shows the comparison between solutions when the solution no medicine is used (just the dynamical system solution is plotted) with when two medications exist (optimal regular control problem solution is plotted). It is observed that modeling the problem just the dynamic system gives a solution to myofibroblast density that never vanishes and therefore apoptosis will happen while the solution for optimal regulator problem decreases and vanishes after almost 300 days. The numerical result in Fig.  10 this verifies that the authors guest realistic assumptions in changing the problem modeling from just a dynamic system to an optimal regulator control problem are correct.
(v) *Convergence of the dynamical system and convergence of the solution of optimal control problem*
  Tables 2 and 4 shows the absolute error of the dynamical system and the optimal control solution. It is observed that the absolute error is decreased when both degrees of freedom and the number of iterations are increased. The absolute error approximation of myofibroblast density with the Lagrangian interpolation polynomials of orders 16, 24, and 32 are calculated. From Tables 2, 3 and 4 wan can see that even for $N=16$ degrees of freedom solutions for both $\hat{m}(t)$ and $\hat{m}^*(t)$ are accurate up to 7 decimal points. Numerical results are plotted in Figs. 11 and 12.

**Table 2:** The absolute error for $|\hat{m}_N(t)-\hat{m}_{N^\prime }(t)|$ where $N=32$, $N^\prime =16$ and 24, for the number of iterations from 4000 to 10000.

Convergent for dynamical system solutions					
Iteration	Norm	Error	Iteration	Norm	Error
4000	$|\hat{m}_{32}(t)-\hat{m}_{24}(t)|$	$8.83\times 10^{-5}$	4000	$|\hat{m}_{32}(t)-\hat{m}_{16}(t)|$	0.00017
7000	$|\hat{m}_{32}(t)-\hat{m}_{24}(t)|$	$6.05\times 10^{-7}$	7000	$|\hat{m}_{32}(t)-\hat{m}_{16}(t)|$	$1.21\times 10^{-6}$
10000	$|\hat{m}_{32}(t)-\hat{m}_{24}(t)|$	$4.15\times 10^{-9}$	10000	$|\hat{m}_{32}(t)-\hat{m}_{16}(t)|$	$8.31\times 10^{-9}$

**Table 3:** Solutions for the optimal regulator control problem for Eqs. (41), 42) with initial and boundary conditions (6) for $N=16, 24$, and 32 at times $t=50, 100, 150$, and 250. Myofibroblast densities are computed for the center point of the region **D** ( $x=y=0.45$).

	$t=50$	$t=100$	$t=150$	$t=250$
$\hat{m}^*_{16}(t)$	0.00782252	0.007199078	0.006625359	0.00561154
$\hat{m}^*_{24}(t)$	0.007822518	0.007199071	0.00662535	0.00561152
$\hat{m}^*_{32}(t)$	0.007822517	0.007199079	0.00662535	0.00561153

**Table 4:** The absolute error for myofibroblast density $|\hat{m}^*_N(t)-\hat{m}^*_{N^\prime }(t)|$ while optimal control solution is solved when $N=32$, $N^\prime =16$ and 24, for the number of iterations from 4000 to 10000.

Convergent for optimal regulator control problem solutions					
Iteration	Norm	Error	Iteration	Norm	Error
4000	$|\hat{m}^*_{32}(t)-\hat{m}^*_{24}(t)|$	$8.853\times 10^{-5}$	4000	$|\hat{m}^*_{32}(t)-\hat{m}^*_{16}(t)|$	0.00017
7000	$|\hat{m}^*_{32}(t)-\hat{m}^*_{24}(t)|$	$6.0608 \times 10^{-7}$	7000	$|\hat{m}^*_{32}(t)-\hat{m}^*_{16}(t)|$	$1.2121\times 10^{-6}$
10000	$|\hat{m}^*_{32}(t)-\hat{m}^*_{24}(t)|$	$4.14908\times 10^{-9}$	10000	$|\hat{m}^*_{32}(t)-\hat{m}^*_{16}(t)|$	$8.2981\times 10^{-9}$

## Discussion and conclusions

IPF is a chronic progressive disease of unknown etiology with approximately 5000 new cases per year and 5-year survival. This rate of incidence and mortality is higher than many other cancers. Furthermore, there is no proven effective treatment for IPF^1,33^. In this article, the homogenized diffusion equation is used to describe the space of lung alveoli. For the first time, we have proposed a mathematical optimal control problem with two control for the treatment of IPF. Anti-TGF-$\beta$ and anti-PDGF medicines in myofibroblast diffusion are controlled successfully. First, the dynamical system of myofibroblast diffusion is solved by Legendre spectral method, and it is shown that using the spectral approximation with 32 nodes can give the proper solution (see Fig. 5). Hence myofibroblasts resist apoptosis in response to serious injury, and persistent repairing leads to tissue remodeling and fibrosis formation. This means that without medication can not expect to cure the disease. Even without any specific strategy, if we give some medication to the patient, myofibroblast density will not vanish (see Fig. 6). In Fig. 6, we showed that with the change of $\eta _T(t)$ and $\eta _G(t)$ in the dynamic system, we can see the reduction of myofibroblasts density but it never vanishes, and it never the cure diseases. Some researchers^5,7–9^ use just the dynamical system and claim that by adjusting the medication doses can cure diseases and control fibrosis. Here, we show that in this manner there is no way to force the myofibroblast density to vanish and it never removes. For this reason, the authors model the problem as an optimal regulator problem with two controls as anti-TGF-${\beta }$ and anti-PDGF medicines. Here, we improve one of the models of Bahram Yazdroudi and Malek^21^ to achieve the goals presented in this paper. It is observed that the control functions (anti-TGF-${\beta }$ and anti-PDGF) decrease and then remain zero after almost 300 days. Hence, in repair tissue, fibroblasts vanish through apoptosis, and no formation of fibrosis tissue happens. The medicines are prescribed from a certain dose, then decrease and vanish over time. With this strategy, there is no need to prescribe medicines during the last days of the patient take cure duration and the disease will be cured. When comparing two strategies (without and with control) for myofibroblast density, we consider that when there is no control, the myofibroblast increase and then remain constant (failed apoptosis). When there is control, after almost 300 days of controlling both anti-TGF-${\beta }$ and anti-PDGF, the myofibroblast density decreases and then vanishes. For example, the medicine *Pirfenidone* has been identified as an anti-TGF-$\beta$^34^ (a TGF-$\beta$ inhibitor that blocks TGF-$\beta$ activity) and *Imatinib* as an anti-PDGF therapy, (a PDGF inhibitor that blocks PDGF activity). Numerical results in this paper, corroborate the idea of vanishing myofibroblast density by medication. To control myofibroblast proliferation, myofibroblast contraction, and apoptosis^34,35^, prescription of both anti-TGF-$\beta$ and anti-PDGF medicines including antibodies is proposed. By this strategy, apoptosis and reduced myofibroblasts density prevent the formation of collagen in ECM^34^. It is observed that with the passage of time and taking medication, the myofibroblast density becomes zero after about 300 days. The patient needs both medicines anti-TGF-$\beta$ for about 155 days and anti-PDGF for about 270 days to treat fibrosis. Here, in objective functional for the optimal control problem, the dosage of treatment through the use of anti-TGF-$\beta$ and anti-PDGF medicines are the same. In further strategy, the dosage of anti-TGF-$\beta$ and anti-PDGF medicines can be assumed to be different. In the further works the authors are going to discuss the effect of anti-IL13, in TGF-$\beta$ and fibroblast, and the effect of anti-$T_{\alpha }$, in M1 and M2.

## Data Availability

All data generated or analyzed during this study are included in this published article.
